# Membrane stability of sickle erythrocytes incubated in extracts of three medicinal plants: *Anacardium occidentale, Psidium guajava, and Terminalia catappa*

**DOI:** 10.4103/0973-1296.80669

**Published:** 2011

**Authors:** Paul Chidoka Chikezie, Augustine Amadikwa Uwakwe

**Affiliations:** *Department of Biochemistry, Imo State University, Owerri, Imo State, Nigeria*; 1 *Department of Biochemistry, University of Port-Harcourt, Port-Harcourt, Rivers State, Nigeria*

**Keywords:** *A. occidentale*, Erythrocytes, *P. guajava*, Saline, *T. catappa*

## Abstract

**Background:**

Many reports showed that medicinal plant extracts cause alterations on the shape and physiology of erythrocytes.

**Objective:**

The present study seeks to ascertain the osmotic stability of sickle erythrocytes incubated in aqueous extracts of *Anacardium occidentale, Psidium guajava,* and *Terminalia catappa*.

**Materials and Methods:**

The fraction of erythrocytes lysed when suspended in saline solution of varying concentrations was investigated by spectrophotometric method. The percentage hemolysis of erythrocytes in the control and test samples showed a sigmoidal relationship with increasing concentrations of saline solution. Membrane stability was ascertained as mean corpuscular fragility (MCF) index of erythrocytes incubated in 400 and 800 mg/dL aqueous concentrations of the three plant extracts.

**Results:**

The two experimental concentrations of *P. guajava* and *T. catappa* protected the erythrocytes against osmotic stress, as evidenced by decreases in the values of MCF compared with the control sample (*P* < 0.05). However, 800 mg/dL of *A. occidentale* promoted significant (*P* < 0.05) distabilization of sickle erythrocytes.

**Conclusion:**

Whereas the two experimental concentrations of aqueous extracts of *P. guajava* and *T. catappa* stabilized erythrocyte membrane, higher concentration (800 mg/dL) of *A. occidentale* exhibited no membrane protective effect.

## INTRODUCTION

Sickle cell anemia (SCA) is highly frequentin sub-Saharan Africa, the Middle East and Mediterranean areas,the Indian subcontinent, the Caribbean, and South America.[1–3] The sickle erythrocyte hemoglobin (HbS) disorder is caused by a point mutation affecting the coding sequence ofthe β-globin gene, causing a substitution of glutamic acidby valine at the sixth position of β-globins.[4] This amino acid substitution leads to a drasticreduction in the solubility (gelation) of deoxy-HbS molecules. Underlow oxygen tension, deoxy-HbS molecules polymerize, causing the formation of rigid and sickled erythrocytes. The deformity of the sickled erythrocyte results in their shortened survival since they become vulnerable to lysis as they penetrate the interstices of the splenic sinusoids and hence severe hemolytic anemia ensues with hemoglobin values ranging from 6 to 10 g/L.[5,6] Thehomozygous state of SCA is associated with complications anda reduced life expectancy.[4,7]

Some medicinal plant extracts have been demonstrated by *in vitro* investigations to reduce polymerization of HbS molecules[8,9] and have been established to serve as potential chemotherapeutic preparations for alleviation and management of SCA.[10–12] For these agents to exert therapeutic benefit, they come in direct contact and interact with membrane architectural components and cellular processes required for erythrocyte functional and structural integrity. Therefore, further studies should be carried out to ascertain the stability and functionality of the erythrocytes in the presence of these antipolymerization/sickling agents.

Osmotic fragility index is a measure of the capacity of erythrocytes to withstand osmotic stress.[13] The test is clinically useful for diagnosis of hereditary spherocytosis[14] and to ascertaining the stability and functionality of erythrocyte plasma membrane.[15,16] The present study seeks to ascertain the stability of sickle erythrocyte when subjected to osmotic stress and incubated in aqueous extracts of *Anacardium occidentale, Psidium guajava*, and *Terminalia catappa*.

## MATERIALS AND METHODS

### Collection plant specimens

Fresh samples of *A. occidentale*, *P. guajava*, and *T. catappa* leaves were harvested between July and August 2010, from trees within the environment of Imo State University, Owerri, Nigeria. The plant specimen were identified and authenticated by Dr. F. N. Mbagwu at the Herbarium of the Department of Plant Science and Biotechnology. A voucher specimen was deposited at the Herbarium for reference purposes.

### Preparation of aqueous extract of plant specimens

The samples were washed under continuous current of distilled water for 15 min and air dried at room temperature for 60 min. The separate leaves were dried for 5 h in an oven at 65°C to become crispy, and ground with ceramic mortar and pestle. Two grams each, of the pulverized specimens was suspended in 1.0 dL of distilled water and allowed to stand for 6 h at 37°C. The aqueous extracts (2 g/mL) of *A. occidentale*, *P. guajava*, and *T. catappa* leaves were obtained by filtration with Whatman No. 2 filter paper. The prepared extracts were kept at 4°C in a refrigerator for at least 24 h before subsequent tests. Dilution equivalents of 400 and 800 mg/dL the aqueous extracts were used for osmotic fragility test.

### Collection of blood samples/preparation of erythrocyte hemolysate

Five milliliters (5.0 mL) of human venous blood samples of HbSS genotype were collected by venipuncture and stored in Na_2_EDTA anticoagulant tubes. The blood samples were obtained between July and August, 2010, from 9 male volunteers (59–79 kg) between the age bracket of 21–34 years attending clinics at the Federal Medical Center (FMC), Imo State University Teaching Hospital (IMSUTH), Orlu, St. John Clinic/Medical Diagnostic Laboratories, Avigram Medical Diagnostic Laboratories, and Qualitech Medical Diagnostic Laboratories. These centers are located in Owerri, Imo State, Nigeria. The Institutional Review Board of the Department of Biochemistry, Imo State University, Owerri, Nigeria, granted approval for this study and all volunteers involved signed an informed consent form. This study was in accordance with the ethical principles that have their origins in the Declaration of Helsinki.

Within 2 h of collection of blood samples, portions of 1.0 mL of the samples were introduced into centrifuge test tubes containing 3.0 mL of buffer solution pH = 7.4: 250 mM tris (hydroxyl methyl) amino ethane-HCl (Tris–HCl)/140 mM NaCl/1.0 mM MgCl_2_/10 mM glucose). The erythrocytes were separated from plasma by centrifugation at 1200 × *g* for 10 min and washed 3 times by the same centrifugation method with the buffer solution. The erythrocytes were finally re-suspended in 1.0 mL of this buffer and stored at 37°C.[17]

### Erythrocyte osmotic fragility tests

Determination of erythrocyte osmotic fragility was carried out based on the method described by Dewey *et al*,[18] with minor modifications as reported by Chikezie.[9] The fraction of erythrocytes lysed when suspended in saline solution of varying concentrations was investigated by spectrophotometric method. A stock of phosphate buffered saline solution, osmotically equivalent to 0.9 g/dL NaCl was prepared as follows: NaCl (90 g), Na_2_HPO_4_˙2H_2_O (17.1 g), and NaH_2_PO_4_˙2H_2_O (2.43 g) were dissolved in 1 L of distilled water. A final volume of 50 mL of saline solution of dilution equivalents, 0.9, 0.7, 0.5, 0.3, and 0.2 g/dL were prepared according to Chikezie *et al*.[19]

Five milliliters (5.0 mL) of saline solution (0.9–0.2 g/dL NaCl) was introduced into corresponding 5 test tubes and 5.0 mL of distilled water was added to the sixth test tube. A 0.5 mL of each plant extract solution of varying concentrations as specified was delivered into each of the given set of test tubes (1–6). To each test tube, 0.05 mL of the erythrocyte suspension was added and mixed thoroughly by inverting the tubes several times. For the control experiment, the same procedure was repeated but devoid of solution of the plant extracts. The suspensions were allowed to stand for 30 min at room temperature (24–27°C) after which the contents were centrifuged at 1200 rpm for 15 min. The relative amount of hemoglobin released into the supernatant was determined with the use of a spectrophotometer (Digital Blood Analyzer^®^; SPECTRONIC 20, Labtech) at maximum wavelength (λ_max_) of 540 nm. The buffered sodium chloride solution and distilled water served as blank and 100% lysis point, respectively.

### Evaluation of percentage hemolysis and stabilization of erythrocytes

The quotient of absorbance of the content of individual corresponding test tubes (1–5) and the sixth test tube were obtained and multiplied by a factor of 100. The range of values represented the percentage of erythrocyte lysis at each saline concentration (0.9–0.2 g/dL NaCl), respectively. The corresponding concentration of saline solution (NaCl g/L) that caused 50% lysis of erythrocytes was the mean corpuscular fragility (MCF) index.[16,18] The MCF values were interpolated from the cumulative erythrocyte osmotic fragility curves obtained by plotting the percentage lysis against saline concentrations.

The relative capacity of the plant extracts to stabilize or disrupt erythrocyte membrane was evaluated as percentage of the quotient of the difference between the MCF values of the test and control sample to the control sample.[9]

$$\mathrm{Thus},\;\%\;\mathrm{Stability}\;=\;\frac{(MCF\;contorl\;–\;MCF\;test)\;100}{MCF\;contorl}$$

### Statistical analysis

The data of percentage hemolysis were analyzed by Student’s *t* test as described by Pearson and Hartley.[20] Mean *P* < 0.05 was considered significant.

## RESULTS

A broad survey of Table 1 shows that within the experimental concentrations ([NaCl] = 0.9–0.2 g/dL), the percentage of hemolysed sickle erythrocytes increased with decreasing concentrations of saline solution. In the control and test analyses, maximum level of hemolysis (100%) occurred when the erythrocytes were suspended in distilled water.

The percentage of hemolysis of the control and test samples showed a sigmoidal relationship with increasing concentrations of saline solution (not shown). Table 1 showed that at 0.9 g/dL saline solution, sickle erythrocytes incubated in 800 mg/dL concentration of *A. occidentale* exhibited minimal level of hemolysis (0.93%±1.31%) and was not significantly different (*P* < 0.05) from the control sample (1.88%±0.80%). Aqueous extracts of the three medicinal plants at the two experimental concentrations (400 and 800 mg/dL) caused significant (*P* < 0.05) level of hemolysis at saline concentrations of 0.5 and 0.2 g/dL. On the contrary, specifically at the saline concentration of 0.7 g/dL, the capacity of the three plant extracts to cause hemolysis of sickle erythrocytes was not significant (*P* > 0.05) except erythrocytes that were incubated in 400 and 800 mg/dL of *A. occidentale* and *T. catappa*, respectively. In the presence of aqueous extracts of the three medicinal plants, except 800 mg/dL concentration of *A. occidentale*, the osmotic fragility curves (not shown) derived from data in Table 1 were significantly shifted to left.

**Table 1:** Percentage hemolysis of human sickle erythrocytes incubated in aqueous extracts of *A. occidentale, P. guajava*, and *T. catappa*

Equivalent NaCl (g/dL)	Hemolysis (%)					
	0.9	0.7	0.5	0.3	0.2	0.0
Control; n = 9	1.88 ± 0.80	3.53 ± 0.99	39.34 ± 5.77	67.75 ± 6.52	76.34 ± 5.68	100.00 ± 0.00
[*A. occidentale*]; n = 9						
400 mg/dL	4.72 ± 2.57^a^	7.68 ± 2.85^a^	29.96 ± 5.43^a^	55.80 ± 5.76^a^	91.37 ± 4.47^a^	100.00 ± 0.00
800 mg/dL	0.93 ± 1.31	3.88 ± 2.85	60.35 ± 3.37^a^	67.89 ± 7.23	83.65 ± 4.65^a^	100.00 ± 0.00
[*P. guajava*]; n = 9						
400 mg/dL	3.98 ± 1.21^a^	4.74 ± 1.38	6.28 ± 1.56^a^	39.57 ± 5.98^a^	82.06 ± 5.39^a^	100.00 ± 0.00
800 mg/dL	3.95 ± 1.60^a^	4.77 ± 1.79	10.12 ± 4.30^a^	59.03 ± 4.90^a^	68.05 ± 7.08^a^	100.00 ± 0.00
[*T. catappa*]; n = 9						
400 mg/dL	2.91 ± 1.72	4.57 ± 1.30	8.24 ± 2.81^a^	53.84 ± 5.24^a^	85.91 ± 5.15^a^	100.00 ± 0.00
800 mg/dL	4.15 ± 3.89^a^	7.09 ± 5.57^a^	14.30 ± 6.08^a^	68.66 ± 7.06	87.68 ± 1.28^a^	100.00 ± 0.00

Values are means of 9 determinations ± S.D

a Difference in hemolysis is significant (*P* < 0.05).

The MCF values of sickle erythrocyte incubated in *P. guajava* and *T. catappa* extracts were not significantly different (*P* > 0.05) from the control sample. An overview of Table 2 showed that aqueous extracts of *P. guajava* and *T. catappa* exhibited no significant (*P* > 0.05) stabilizing effect on sickle erythrocytes at 400 and 800 mg/dL. However, the capacity of *P. guajava* and *T. catappa* to stabilize the eythrocytes diminished with increasing concentration of the two plant extracts. Whereas 400 mg/dL aqueous extract of *A. occidentale* engendered erythrocyte stabilization, higher concentration elicited destabilization ([*A. occidentale*] = 800 mg/dL; MCF = 0.543±1.12; destabilization = 28.67%; P > 0.05).

**Table 2:** Human sickle erythrocytes mean corpuscular fragility and stability (%) in the presence of *A. occidentale, P. guajava* and *T. catappa*

[Extract] (mg/dL)	*A. occidentale*		*P. guajava*		*T. catappa*	
	MCF (g/dL)	Stability	MCF (g/dL)	Stability	MCF (g/dL)	Stability
(control)	0.422 ± 0.007	-	0.422 ± 0.007	-	0.422 ± 0.007	-
400	0.342 ± 0.010	18.96^s^	0.279 ± 0.011	33.89^s^	0.320 ± 0.006	24.17^s^
800	0.543 ± 0.007	28.67^d^	0.337 ± 0.009	20.14^s^	0.372 ± 0.013	11.84^s^

MCF values are means of 9 determinations ± S.D (Microsoft Office Excel, 2007 version): Difference in MCF values is significant (*P* < 0.05)

d Percentage of membrane destabilization

s Percentage of membrane stabilization

## DISCUSSION

In this study, the pattern of hemolysis of sickle erythrocytes suspended in varying concentrations of saline solution conformed to previous findings of erythrocytes obtained from bream *Abramis brama* (L) from Zalew Szczecinski,[21] allophenic mice,[18] birds,[22] human,[8,9,19] and among amphibians, reptiles, birds, and mammals.[23] In addition, the reports of this study showed that aqueous extracts of *A. occidentale*, *P. guajava*, and *T. catappa* interfered with erythrocyte membrane integrity and stability in conformity to findings reported elsewhere.[24]

To a large extent, the present investigation has also demonstrated the capability of the three plant extracts to stabilize erythrocyte membrane structures, which was an indication of reduced hemolysis in induced hypotonic-stress condition. Contrary, a higher concentration of *A. occidentale* (800 mg/dL) caused membrane destabilization, which may not be unconnected with relatively increased level of tannins in *A. occidentale*, a chaotropic agent that has been reported to compromise erythrocyte membrane integrity.[25] Similarly, the dwindling levels of membrane stability with increasing concentrations of *A. occidentale*, *P. guajava*, and *T. catappa* [Table 2] suggest a corresponding increasing level and capacity of certain chaotropic agents in the crude extracts to promote membrane destabilization. Therefore, it is envisaged that the elimination of these membrane destabilization agents by further purification of the crude plant extracts could serve to increase the potency of these plant products to stabilize erythrocyte membrane.

Sickle erythrocytes are susceptible to endogenous free radical-mediated oxidative damage that correlates with the proportion of irreversibly sickled erythrocytes.[26] In addition, Van Kuijk *et al*[27] and Tamer *et al*,[28] reported that higher levels of free radicals in human sickle erythrocytes was associated with increased tendency of diminished mechanical and osmotic stability. Therefore, accumulation of oxidant contributes to accelerated damage of sickle erythrocyte membrane and senescence of these cells.[26] Several research findings have been conducted to establish that free radicals can be blocked and/or scavenged.[27–31] Furthermore, reports by Benavente-García *et al*,[32] Okwu,[33] Miliauskas *et al*,[34] and Kumar *et al*[35] showed that lipid peroxidation and subsequent biomembrane damage can be inhibited by flavonoids, which serve as strong radical scavengers and singlet oxygen quenchers.

Although the present study did not establish the mechanism and the active principles of the three extracts that are responsible for membrane stabilizing effect on sickle erythrocytes, earlier researchers have implicated the flavonoids, triterpenoids, and host of other secondary plant metabolites/constituents as the major contributors to membrane stabilization.[36–38]
